# The effect of prior hip arthroscopy on outcomes of total hip arthroplasty: a systematic review of comparative studies

**DOI:** 10.1186/s12891-026-10094-7

**Published:** 2026-06-16

**Authors:** Rémy Coulomb, Férid Harrar, Youssef Jamaleddine, Redha Belal, Pascal Kouyoumdjian

**Affiliations:** 1 Centre Hospitalier Universitaire de Nîmes, Nîmes, France; 2 University Mechanical and Civil Engineering Laboratory UMR 5508 CNRS-UMCC 048, 163 rue Auguste Broussonnet, Montpellier, 34090 France; 3 Centre Hospitalier d’Arles, Arles, France; 4 Department of Orthopedic Surgery, Lebanese American University Medical Center-Rizk Hospital, Beirut, Lebanon

**Keywords:** Conservative surgery, Total hip arthroplasty, Femoroacetabular impingement, Complication, Outcome, Conversion

## Abstract

**Background:**

Hip arthroscopy is increasingly used to treat femoroacetabular impingement, labral tears, and related disorders in young and active patients. As its indications have expanded, more patients subsequently undergo total hip arthroplasty (THA). Whether prior ipsilateral hip arthroscopy adversely affects the results of THA remains unclear. This systematic review assessed whether previous hip arthroscopy influences (1) perioperative findings, (2) postoperative complications and revision-related events, and (3) postoperative functional outcomes after THA.

**Methods:**

A systematic review was performed according to PRISMA principles. PubMed, ScienceDirect, Google Scholar, Embase and Cochrane Library were searched for English-language comparative studies published between 2010 and 2025 evaluating THA after prior ipsilateral hip arthroscopy versus THA without prior arthroscopy. Extracted data included study design, patient characteristics, perioperative findings, postoperative complications, revision-related events, and patient-reported outcomes. Methodological quality was assessed using MINORS. Because of substantial heterogeneity in study design, follow-up, and reported outcomes, a narrative synthesis was performed.

**Results:**

Eighteen retrospective comparative studies were included, comprising 28,477 arthroplasty patients, including 8,437 with prior hip arthroscopy. Perioperative differences were generally limited, although several studies reported longer operative time and, less consistently, greater blood loss after prior arthroscopy. Most studies found no clear differences in infection, fracture, leg-length discrepancy, or heterotopic ossification. Evidence regarding revision-related outcomes was heterogeneous: several matched cohorts and database studies reported higher rates of revision, loosening, dislocation, reoperation, or infection, particularly when THA was performed within 1 year of arthroscopy or after arthroscopy in osteoarthritic hips, whereas other studies found no increase. Functional outcomes improved substantially in both groups, although some studies reported slightly lower postoperative scores or satisfaction after prior arthroscopy.

**Conclusion:**

Prior ipsilateral hip arthroscopy does not appear to compromise subsequent THA in most patients. Perioperative differences were generally limited, and functional improvement after THA remained substantial overall. Evidence regarding postoperative complications and revision-related events was heterogeneous, with some studies reporting higher risk in selected patients, particularly when THA was performed within 1 year of arthroscopy or after arthroscopy in already degenerative hips.

## Introduction

Total hip arthroplasty (THA) is a reliable treatment for end-stage hip degeneration, providing durable pain relief and functional restoration in appropriately selected patients [1]. In parallel, hip arthroscopy has expanded substantially and is now widely used to treat femoroacetabular impingement (FAI), labral tears, and selected chondrolabral disorders in younger and more active patients [2]. Although arthroscopy may delay joint arthroplasty in some cases, conversion to THA remains common in symptomatic degenerative hips, with reported rates ranging from 9.5% to 50% [3]. Older age and smaller joint-space width also appear to increase the risk of conversion after arthroscopy [4].

As the number of arthroscopic procedures has increased, arthroplasty surgeons are more frequently encountering patients who undergo THA after prior ipsilateral hip arthroscopy. Whether previous arthroscopy alters soft-tissue planes, capsular integrity, bone stock, or periarticular scarring sufficiently to influence subsequent THA remains debated. Published comparative studies have reported conflicting findings, with some showing higher complication or revision rates and others finding no clinically meaningful difference in perioperative measures or postoperative function [5–22].

The aim of this systematic review was to assess whether prior ipsilateral hip arthroscopy influences the results of subsequent THA. More specifically, we examined whether previous arthroscopy affects: (1) perioperative findings at the time of THA; (2) postoperative complications and revision-related events; and (3) postoperative functional outcomes.

## Materials and methods

This systematic review was conducted in accordance with PRISMA principles [23]. PubMed, ScienceDirect, Google Scholar, Embase and Cochrane Library were searched for English-language studies published between January 2010 and April 2025. The following keywords were used and combined with the Boolean operators “AND” and “OR” to identify comparative studies evaluating total hip arthroplasty after prior hip arthroscopy: (“hip arthroscopy”) AND (“total hip arthroplasty” OR “total hip replacement”) AND (“outcomes” OR “complications” OR “revision” OR “comparison”).

Eligibility criteria were defined according to the PICOS framework. The population comprised patients undergoing hip arthroplasty for degenerative hip disease. The intervention group included patients with prior ipsilateral hip arthroscopy, and the comparator group included patients undergoing arthroplasty without prior ipsilateral arthroscopy. Eligible studies were required to report at least one of the following outcomes: perioperative parameters, postoperative complications, implant-related events, revision-related outcomes, or functional outcomes. Comparative retrospective or prospective studies were included. Studies combining arthroscopic and open prior hip surgery without separate analysis, non-comparative case series, case reports, conference abstracts, literature reviews, and studies focused on arthroplasty after fracture, tumour, or infection were excluded.

**Fig. 1:**
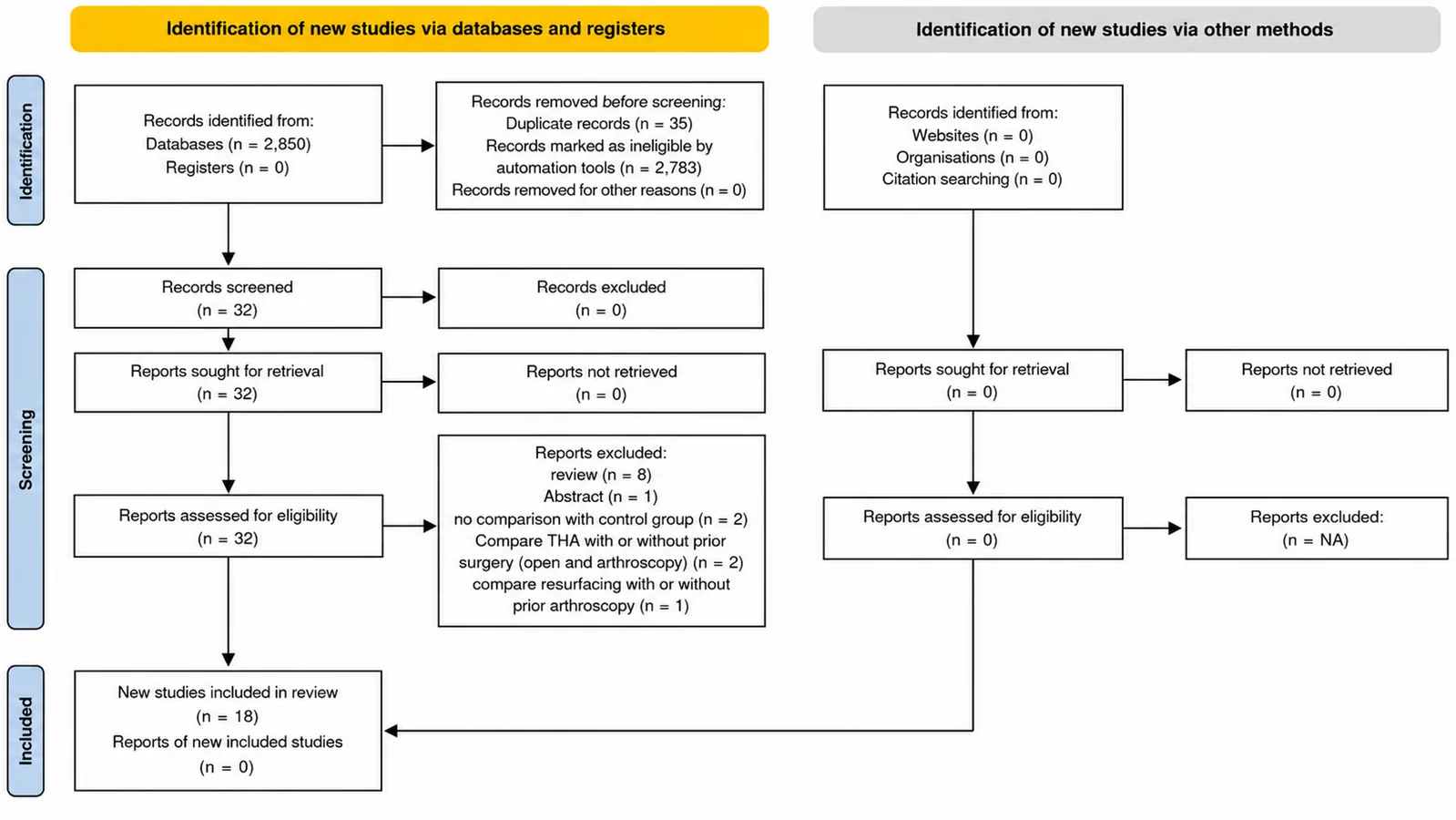
PRISMA flow chart showing identification, screening, eligibility assessment, and inclusion of studies in the systematic review

Study selection was performed in two stages, screening of titles and abstracts, followed by full-text review of potentially eligible articles. Eligibility of the included studies was determined by two reviewers independently. Disagreements when present were discussed and resolved by a third senior independent author. After removal of duplicates and clearly irrelevant records, 32 articles underwent full-text assessment. Fourteen studies were excluded after detailed review: 8 literature reviews, 1 conference abstract, 2 studies without a control group, 2 studies comparing THA after prior hip surgery without isolating arthroscopy, and 1 resurfacing study. Eighteen comparative studies were ultimately included in the qualitative synthesis (Fig. 1).

Data extraction was performed using a standardized form. The following variables were collected: study design, level of evidence, number of patients, sex ratio, age, body mass index, follow-up, interval between arthroscopy and THA, indication for arthroscopy, indication for THA, surgical approach, perioperative findings, postoperative complications, revision-related events, and patient-reported outcome measures.

Methodological quality was assessed with the Methodological Index for Non-Randomized Studies (MINORS) [24]. Because all included studies were non-randomized and substantial heterogeneity was present across study design, cohorts, follow-up, and reported outcomes, no meta-analysis was performed. Although several outcomes such as revision, reoperation, dislocation, and infection were reported by multiple studies, exploratory pooling was not performed because these endpoints were defined and measured inconsistently across studies. For example, some studies reported all-cause revision, whereas others reported aseptic revision, reoperation, loosening, dislocation, infection, or composite complication outcomes over different follow-up windows. In addition, the included studies differed substantially in database versus institutional design, matching strategy, arthroscopy indication, interval between arthroscopy and THA, surgical approach, and duration of follow-up. Therefore, a narrative synthesis was considered more appropriate than a pooled estimate that could be clinically misleading. Results were therefore synthesized narratively. Ethical approval was not required because this study was based exclusively on previously published data.

## Results

### Study selection, characteristics, and methodological quality

All 18 included studies were retrospective comparative studies and therefore corresponded to level-III evidence. Together, they included 28,477 arthroplasty patients, of whom 8,437 had undergone prior ipsilateral hip arthroscopy. Minimum follow-up thresholds ranged from 90 days to 5 years, and mean follow-up, when reported, was generally between 2 and 5 years. When specified, the interval between arthroscopy and THA was usually short, ranging from approximately 1.1 to 3.1 years in most series. Study characteristics are summarized in Table 1.

**Table 1:** Study characteristics. A+/A-: Prior arthroscopy/ No prior arthroscopy; M/F: male/female; BMI: body mass index; NA: not specified. Sex-ratio entries are reported as group-specific male/female counts for Arthro+/Arthro- when explicitly available; otherwise NA. Follow-up values are reported as study-reported mean follow-up when available; database studies and event-driven analyses are reported using the study outcome window or minimum required follow-up as stated in the source. A ≥ symbol indicates minimum required follow-up only. In Parker et al. and Konopka et al., only the THA subgroup was extracted from the original mixed THA/HRA studies. In Parker et al., the reported 4.5/6.0-year values reflect the overall mixed arthroplasty case/control cohorts because THA-only follow-up was not separately reported

Study	Level	Patients A+/A-	Sex ratio (M-F) A+/A-	Mean age (years) A+/A-	BMI A+/A-	Follow-up / outcome window	Delay arthroscopy-to-THA (years)
Parker et al. [8]	III	66 22/44	NA	NA	NA	4.5/6.0	1.9
Bolarinwa et al. [5]	III	11,061 110/10,951	NA	NA	NA	3-year outcomes	NA
Charles et al. [12]	III	78 39/39	14–25/14–25	42.4/43.8	31.6/31.3	4.4/4.2	NA
Haynes et al. [9]	III	174 58/116	19–39/38–78	48/49	28/28.6	3.2/3.5	2.3
Hoeltzermann et al. [14]	III	66 33/33	NA	52.8/55	27.7/27.1	3.8/3.3	1.36
Haughom et al. [6]	III	126 42/84	18–24/36–48	51.2/52.9	27.5/28.9	3	1.8
Konopka et al. [16]	III	129 43/86	NA	51.8/54	26/26.2	2	2.4
Lemme et al. [7]	III	3880 1940/1940	NA	NA	NA	4-year outcomes	3.1
Lindman et al. [10]	III	675 135/540	84 − 51/352 − 188	51/52	27/27	1	2.3
Malahias et al. [11]	III	5200 2600/2600	NA	NA	NA	2-year outcomes	1.12
Zingg et al. [15]	III	54 18/36	NA	46.3/50.4	23.9/24.7	2	NA
Pate et al. [21]	III	71 38/33	14–24/10–23	52/58.4	28.7/29.7	≥ 0.25	NA
Perets et al. [13]	III	70 35/35	15–20/15–20	53.4/53.7	29/28.4	3.4	1.55
Rosinsky et al. [17]	III	123 34/89	NA	55.6/53.5	29.2/29	≥ 5	1.47
Ross et al. [18]	III	6312; 3156/3156	NA	54.4/54.4	NA	2-year outcomes	2.17
Vovos et al. [19]	III	190 95/95	43–52/43–52	53/53	29.8/28.8	2	2.4
Jain et al. [20]	III	81 18/63	8–10/32 − 31	43/50	NA	2.2/2.2	1.7
Stock et al. [22]	III	121 21/100	NA	NA	NA	NA	NA

Methodological quality was acceptable overall. Based on the MINORS totals shown in Table 2, 11 studies were of good methodological quality (17 to 24 /24) and 7 were of moderate quality (9 to 16 /24); no study was classified as low quality.

**Table 2:** MINORS methodological assessment. MINORS: Methodological Index for Non-Randomized Studies. Each item was scored 0 (not reported), 1 (reported but inadequate), or 2 (reported adequately)

Study	Aim	Consecutive patients	Prospective data	Endpoints appropriate	Unbiased assessment	Adequate follow-up	Loss to follow-up < 5%	Prospective sample-size calc.	Adequate control group	Contemporary groups	Baseline equivalence	Adequate statistics	Total /24
Parker et al. [8]	2	1	0	2	2	2	0	2	1	2	1	2	17
Bolarinwa et al. [5]	2	1	1	2	2	2	0	0	2	2	1	2	17
Charles et al. [12]	1	1	1	2	1	2	0	0	2	2	1	2	15
Haynes et al. [9]	2	1	1	2	2	2	0	0	2	2	1	2	17
Hoeltzermann et al. [14]	2	1	2	2	2	2	0	2	2	2	1	2	20
Haughom et al. [6]	2	1	1	2	2	2	0	2	2	2	1	2	19
Konopka et al. [16]	2	1	2	2	2	2	0	0	2	2	1	2	18
Lemme et al. [7]	2	1	1	2	1	2	0	0	2	2	1	2	16
Lindman et al. [10]	2	0	1	1	1	2	0	0	2	2	1	2	14
Malahias et al. [11]	2	1	1	1	1	2	0	0	2	2	1	2	15
Zingg et al. [15]	2	2	2	2	2	1	0	0	2	2	1	2	18
Pate et al. [21]	1	2	2	2	1	1	0	0	2	2	1	2	16
Perets et al. [13]	2	0	2	2	2	2	0	2	2	2	1	2	19
Rosinsky et al. [17]	2	0	1	2	2	2	0	2	2	2	2	2	19
Ross et al. [18]	2	1	1	2	1	2	0	0	2	2	0	2	15
Vovos et al. [19]	2	2	1	2	1	2	0	0	2	2	1	2	17
Jain et al. [20]	2	2	1	2	2	2	0	0	2	2	1	2	18
Stock et al. [22]	1	1	1	2	1	1	0	0	2	2	1	2	14

### Indications and surgical approach

Femoroacetabular impingement was the main indication for arthroscopy in most series, sometimes associated with labral tears or tendinopathy. THA was performed predominantly for osteoarthritis. The posterolateral approach was the most frequently reported arthroplasty approach, although several studies used anterior or mixed approaches. Only a minority of studies explicitly stated that both procedures were performed by the same surgeon. These descriptive findings are detailed in Table 3.

**Table 3:** Indications and surgical approach. FAI: femoroacetabular impingement; THA: total hip arthroplasty; NA: not specified

Study	Indication for arthroscopy	Indication for THA	THA approach	Same surgeon
Parker et al. [8]	Mixed	Osteoarthritis	Posterior	NA
Bolarinwa et al. [5]	FAI	Osteoarthritis	NA	No
Charles et al. [12]	FAI	Osteoarthritis	Posterior	Yes
Haynes et al. [9]	FAI + labral lesion	Osteoarthritis	NA	No
Hoeltzermann et al. [14]	FAI	Osteoarthritis	Mixed (20 posterior, 12 anterior, 1 lateral)	No
Haughom et al. [6]	FAI	Osteoarthritis	Mostly posterior; some anterolateral/ Watson-Jones	NA
Konopka et al. [16]	FAI	Osteoarthritis	Posterior	NA
Lemme et al. [7]	NA	NA	NA	NA
Lindman et al. [10]	FAI	NA	NA	NA
Malahias et al. [11]	Osteoarthritis	Osteoarthritis	NA	NA
Zingg et al. [15]	FAI	Osteoarthritis	Minimally invasive anterior	No
Pate et al. [21]	FAI	Osteoarthritis or avascular necrosis	Anterior	Yes
Perets et al. [13]	FAI	Osteoarthritis	Anterior or posterior	Yes
Rosinsky et al. [17]	FAI + labral lesion	Osteoarthritis	Anterior or posterior	No
Ross et al. [18]	NA	Osteoarthritis	NA	NA
Stock et al. [22]	NA	NA	NA	NA
Vovos et al. [19]	FAI + labral lesion + tendinopathy	Osteoarthritis	NA	NA
Jain et al. [20]	Mostly FAI	Osteoarthritis	Posterior	Yes

### Perioperative findings at the time of THA

Six studies reported perioperative measures. Overall, prior arthroscopy did not consistently alter operative conditions, but several signals emerged. Two studies reported significantly longer operative time in the prior-arthroscopy group, whereas one older study reported longer operative time in controls. One study found significantly greater blood loss after prior arthroscopy, but other series did not confirm differences in blood loss, haemoglobin drop, or transfusion rate. Overall, the available evidence suggests that prior arthroscopy may modestly increase technical difficulty in selected cases, but the effect is neither large nor uniform across studies. Perioperative measures are summarized in Table 4.

**Table 4:** Perioperative measures. A+: prior arthroscopy; A-: no prior arthroscopy; NA: not specified

Study	Operative time (minutes)	Blood loss (mL)	Hemoglobin drop (g/dL)	Transfusion	Significant difference
Charles et al. [12]	A + 132.9+/-31.2 A- 136.5+/-39.3 (*p* = 0.678)	A + 616.3+/-276.9 A-554.4+/-275.4 (*p* = 0.314)	A + 4.2+/-1.3 A-4.3+/-1.1 (*p* = 0.774)	A + 2.6% A- 5.1% (*p* = 0.57)	No
Haynes et al. [9]	A + 90.3+/-21.5 A- 88.2+/-31.7 (*p* = 0.647)	NA	NA	NA	No
Hoeltzermann et al. [14]	A + 60.1+/-10.8 A-61.8+/-12.8 (*p* = 0.6)	NA	NA	NA	No
Zingg et al. [15]	A + 118+/-31 A-166+/-39 (*p* = 0.001)	A + 625+/-372 A-693+/-287 (*p* > 0.05)	NA	NA	Operative time
Pate et al. [21]	A + 73.5 A- 60 min (*p* < 0.0001)	A+ 400 A- 275 mL (*p* = 0.009)	NA	1 patient (A+) vs. 0 (A-) (*p* = 1.0)	Operative time Blood loss
Vovos et al. [19]	A + 122.8 A-103.9 (*p* = 0.003)	A + 329.7 A- 274.2 (*p* = 0.32)	NA	A + 13.7% A- 9.5% (*p* = 0.36)	Operative time

### Postoperative complications and revision-related outcomes

Postoperative complications were variably reported across the included studies. Most studies found no significant difference in superficial or deep infection, periprosthetic fracture, leg-length discrepancy, heterotopic ossification, or short-term reoperation. Several studies nevertheless identified higher rates of revision, reoperation, loosening, dislocation, or infection in the prior-arthroscopy cohort, and this signal was strongest in database analyses when THA was performed within 1 year of arthroscopy or when arthroscopy had been performed for osteoarthritis. By contrast, some matched institutional cohorts found no increase in most prosthesis-related complications, while one large database study reported lower 90-day medical complication rates and fewer prolonged opioid claims after THA in patients with prior arthroscopy. Postoperative complications and revision-related outcomes are summarized in Table 5.

**Table 5:** Postoperative complications (reported as percentages). NA: not specified; R: reoperation; L: loosening; B0-1 and B2-4: Brooker grades. Numeric values are percentages unless otherwise specified. P values are shown whenever explicitly reported for the relevant between-group comparison; NA also covers outcomes with no exact p value or no extractable THA-subgroup p value. ‡ In Bolarinwa et al. 2020, the percentages shown correspond to surgical site infection within 3 years and combined dislocation and revision within 3 years, not isolated deep infection or isolated dislocation. In Malahias et al. 2021, univariate Table 3 p values were revision 0.014, PJI 0.010, dislocation 0.012, and aseptic loosening 0.001; multivariate Table 4 p values were all-cause revision 0.012, dislocation 0.073, aseptic loosening < 0.001, and PJI 0.010

Study	Superficial infection Arthro+/Arthro-	Deep infection Arthro+/Arthro-	Reoperation (*R*) or loosening (L) Arthro+/Arthro-	Dislocation Arthro+/Arthro-	Periprosthetic fracture Arthro+/Arthro-	Leg-length discrepancy Arthro+/Arthro-	Heterotopic ossification (Brooker) Arthro+/Arthro-	Significant difference
Parker et al. [8]	NA	0%/0%	R 4.5%/0%	4.5%/2.3%	NA	4.5%/0%	0%/2.3%	No (THA subgroup; mixed-cohort complication *p* = 0.72)
Bolarinwa et al. [5]	NA	3.6%/3.5%‡ (*p* = 0.796)	NA	6.4%/3.7%‡ (*p* = 0.409)	NA	NA	NA	No
Charles et al. [12]	NA	0%/0%	NA	5.1%/0%	NA	NA	B0-1: 94.9%/89.7% B2-4: 5.1%/10.3%	No
Haynes et al. [9]	NA	0%/0.9%	NA	3.4%/0%	NA	NA	NA	No
Hoeltzermann et al. [14]	NA	NA	0%/0%	0%/0%	NA	NA	0%/0%	No
Haughom et al. [6]	NA	2.4%/1.2%	R 7.1%/4.8% (*p* = 0.42)	2.4%/1.2%	0%/1.2%	2.4%/0%	NA	No; overall complications *p* = 0.53
Konopka et al. [16]	NA	NA	2.3%/3.5%	2.3%/1.2%	NA	NA	NA	No
Lemme et al. [7]	NA	2.6%/2.4%	R 2.6%/1.7% (*p* = 0.05) L 1.6%/0.9% (*p* = 0.07)	3.5%/2.3% (*p* = 0.03)	1.0%/1.1%	NA	NA	Reoperation (*p* = 0.05) Dislocation (*p* = 0.03)
Lindman et al. [10]	NA	NA	R 1.5%/3.5%	NA	NA	NA	NA	No; reoperation *p* = 0.3
Malahias et al. [11]	NA	2.9%/1.6% (univ *p* = 0.010)	R 3.4%/2.1% (univ *p* = 0.014; adj *p* = 0.012) L 2.3%/1.0% (univ *p* = 0.001; adj *p* < 0.001)	3.2%/2.3% (univ *p* = 0.012; adj *p* = 0.073)	0.9%/0.8%	NA	NA	Infection Reoperation Loosening Dislocation (univariate only)
Zingg et al. [15]	5.6%/0%	0%/0%	R 5.6%/0%	0%/2.8%	NA	NA	B0-1: 5.6%/22.2% B2-4: 0%/0%	No
Pate et al. [21]	5.3%/0%	0%/0%	R 2.6%/6.1% L 0%/0%	0%/0%	0%/9.1%	NA	2.6%/0%	No
Perets et al. [13]	NA	NA	Future surgery: 11.4%/0% (*p* = 0.114)	NA	NA	NA	NA	Trend toward more complications (Overall complications: 14.3%/0% (*p* = 0.054) ◊ Non significant)
Rosinsky et al. [17]	NA	NA	Revision THA: 14.7%/1.1% (RR 13.09) (*p* = 0.0019)	NA	NA	NA	NA	Revision THA higher in Arthro+; overall complications numerically higher
Ross et al. [18]	NA	0.6%/1.3% (*p* = 0.013)	Aseptic revision: 3.7%/3.1% (*p* = 0.094)	2.9%/2.6% (*p* = 0.633)	0.7%/0.8% (*p* = 0.393)	NA	NA	Lower 90-day medical complications and lower 1-year PJI in Arthro+
Vovos et al. [19]	Wound dehiscence: 5.3%/0% (*p* = 0.023)	SSI: 4.2%/2.1% (*p* = 0.41)	R 12.6%/7.4% (*p* = 0.23)	3.2%/2.1% (*p* = 0.65)	2.1%/0% (*p* = 0.16)	NA	NA	Overall postoperative complications (*p* = 0.007); wound dehiscence
Jain et al. [20]	11.1%/1.6%	0%/0%	NA	0%/0%	NA	NA	NA	No

### Functional outcomes and satisfaction

Ten studies reported postoperative patient-reported outcome measures. In all series, both groups improved substantially after THA. Most studies did not show a significant difference in postoperative Harris Hip Score, WOMAC, UCLA activity score, Oxford Hip Score, Forgotten Joint Score, SF-12, EQ-5D, EQ VAS, hip pain, or satisfaction. OHS reporting was heterogeneous, with different scoring directions across studies. Nevertheless, a subset of cohorts found lower postoperative HHS, OHS, FJS-12, SF-12 physical score, or satisfaction in patients with prior arthroscopy. These differences were generally small and were not reproduced consistently across all studies. Functional outcomes are summarized in Table 6.

**Table 6:** Functional scores

Study	HHS / mHHS Arthro + vs. Arthro-	WOMAC / HOOS / related domains† Arthro + vs. Arthro-	UCLA / LEAS Arthro + vs. Arthro-	OHS* Arthro + vs. Arthro-	FJS-12 Arthro + vs. Arthro-	SF-12 / VR-12 Arthro + vs. Arthro-	Significant difference(s)
Parker et al. [8]	THA only: Pre 47.7 vs. 51.1 (*p* = 0.33) 1-y post 97.0 vs. 92.7 (*p* = 0.63)	NA	NA	Traditional 60 − 12 scale: Pre 46.4 vs. 43.6 (*p* = 0.2) 6-mo post 17.3 vs. 19.5 (*p* = 0.57) 1-y post 24.1 vs. 20.0 (*p* = 0.18)	NA	NA	No (THA subgroup)
Haynes et al. [9]	mHHS: 46.6/87.0 vs. 46.0/88.9 (delta *p* = 0.99)	WOMAC subscales: Pain 41.5/82.1 vs. 44.2/87.3 (*p* = 0.98) Stiffness 37.3/75.3 vs. 43.8/80.1 (*p* = 0.4) Physical Function 44.4/84.9 vs. 46.6/87.0 (*p* = 0.7)	UCLA: 4.92/6.42 vs. 5.07/6.73 (*p* = 0.93)	NA	NA	NA	No
Hoeltzermann et al. [14]	mHHS: 48.0/92.8 vs. 60.3/93.8 (pre *p* = 0.002; post *p* = 0.7)	NA	NA	NA	NA	NA	Pre-op lower in Arthro+ only
Haughom et al. [6]	HHS: 51.7/92.1 vs. 44.9/90.1 (pre *p* = 0.002; post *p* = 0.2)	NA	NA	NA	NA	NA	Pre-op higher in Arthro+ only
Konopka et al. [16]	THA only: NA	THA only: Post-op HOOS: Pain 82 ± 16 vs. 93 ± 9 (*p* = 0.003) Stiffness 85 ± 16 vs. 93 ± 15 (*p* = 0.01) Sports/Rec 71 ± 22 vs. 88 ± 18 (*p* = 0.003) QoL 65 ± 22 vs. 86 ± 11 (*p* < 0.0001) Post-op WOMAC: Pain 86 ± 16 vs. 93 ± 15 (*p* = 0.03) Stiffness 80 ± 21 vs. 88 ± 17 (*p* = 0.05)	THA only: LEAS: no postoperative difference (*p* = 0.18)	NA	NA	THA only: Post-op SF-12: Physical 48 ± 11 vs. 54 ± 6 (*p* = 0.008) Mental: no significant difference (*p* = 0.08)	HOOS pain/stiffness/sports/QoL; WOMAC pain/stiffness; SF-12 physical; lower work satisfaction (*p* = 0.04) and recreation satisfaction (*p* = 0.007)
Lindman et al. [10]	NA	EQ-5D: Pre 0.34 vs. 0.35 (*p* = 0.8) 1-y post 0.81 vs. 0.82 (*p* = 0.9) Delta 0.47 vs. 0.47 (*p* = 0.9) EQ VAS: Pre 52.4 vs. 51.9 (*p* = 0.8) 1-y post 78.3 vs. 78.8 (*p* = 0.8) Delta 25.9 vs. 26.9 (*p* = 0.7) Hip pain: no difference (*p* = 0.7) Satisfaction: 87% vs. 86% (*p* = 0.9)	NA	NA	NA	NA	No
Zingg et al. [15]	NA	Follow-up WOMAC: 1.5 ± 2.3 vs. 1.3 ± 1.6 (*p* = 0.667) (matched-control comparison)	NA	NA	NA	NA	No
Perets et al. [13]	Post-op: 82.6 vs. 90 (*p* = 0.029)	VAS pain: 1.8 ± 2.4 vs. 0.9 ± 1.9 (*p* = 0.053) Satisfaction: 8.1 ± 2.7 vs. 9.3 ± 1.6 (*p* = 0.031)	NA	NA	Post-op: 65.7 vs. 80.3 (*p* = 0.026)	NA	HHS (*p* = 0.029); FJS-12 (*p* = 0.026); satisfaction lower (*p* = 0.031); VAS pain higher, trend only (*p* = 0.053)
Rosinsky et al. [17]	Post-op: 90.7 vs. 88.4 (*p* = 0.559)	VAS pain: 15.36 vs. 11.72 (*p* = 0.155) Satisfaction: 91.43 vs. 87.47 (*p* = 0.264)	NA	NA	Post-op: 83.3 vs. 76.9 (*p* = 0.145)	Post-op VR-12 mental: 59.9 vs. 58.7 (*p* = 0.301) VR-12 physical: 49.4 vs. 49.0 (*p* = 0.808) SF-12 mental: 57.3 vs. 54.7 (*p* = 0.062) SF-12 physical: 48.8 vs. 47.5 (*p* = 0.487)	No
Jain et al. [20]	NA	NA	NA	0–48 scale: Median pre 14 vs. 18.5 Median post 40 vs. 46 (*p* = 0.012) Median change 27 vs. 25	NA	NA	OHS lower after prior arthroscopy (post-op *p* = 0.012)

Table entries are reported as published; some studies reported pre/post values and others only postoperative or follow-up values. *P* values are shown whenever explicitly reported for the comparisons displayed. Parker and Konopka contribute THA subgroups extracted from mixed THA/HRA studies; in Zingg, WOMAC refers to the matched-control follow-up comparison

*HHS* Harris Hip Score, *mHHS *modified Harris Hip Score, *WOMAC *Western Ontario and McMaster Universities Osteoarthritis Index, *HOOS *Hip disability and Osteoarthritis Outcome Score, *UCLA *University of California Los Angeles activity score, *LEAS *Lower Extremity Activity Scale, *OHS *Oxford Hip Score, *FJS-12* Forgotten Joint Score-12, *NA *not specified

* OHS was reported in study-specific formats. Parker et al. used the traditional 60 − 12 scale (lower scores better); Jain et al. used a 0–48 scale (higher scores better)

† WOMAC / HOOS / related domains were grouped in one column

## Discussion

This systematic review addressed a clinically relevant controversy: whether prior ipsilateral hip arthroscopy alters the results of subsequent THA. As hip arthroscopy has expanded in younger and more active patients, arthroplasty surgeons are increasingly confronted with conversion to THA after failed joint-preservation surgery [2–4]. The available literature has remained difficult to interpret because it combines small institutional cohorts with large administrative databases and has produced conflicting conclusions [5–22]. In the present review, overall, the available evidence did not indicate consistently poor outcomes after prior arthroscopy. Rather, prior arthroscopy appeared to define a subgroup in whom THA generally remains effective, but in whom early implant-related complications and selected patient-reported outcomes may be less predictable, particularly when arthroscopy was performed shortly before THA or in already degenerative hips with osteoarthritis. The absence of quantitative pooling reflects substantial heterogeneity across the included studies, including differences in arthroscopy indications, timing to THA, surgical approaches, follow-up duration, and outcome definitions.

Regarding perioperative findings, the available evidence suggests a possible increase in technical complexity, but not a consistent adverse operative impact across all series. This is plausible, because capsulotomy, scar formation, residual deformity, and altered tissue planes may influence exposure and stability at the time of arthroplasty [25]. The perioperative findings were inconsistent, two studies found longer operative time after prior arthroscopy [19, 21], three found no difference [9, 12, 14], and one study reported longer operative time in the control group [15]. Increased blood loss was reported only once [21], while transfusion rates did not differ in studies that assessed this outcome [12, 19, 21]. This pattern suggests that prior arthroscopy may modestly increase the difficulty of THA in selected patients, but that the effect may be context-dependent and may be influenced by the indication for arthroscopy, the amount of residual structural disease, the arthroplasty approach, and surgeon experience.

In contrast to the perioperative findings, the evidence regarding postoperative complications and revision-related outcomes is more consistent in suggesting an increased risk in selected patients. Several studies identified higher rates of selected adverse outcomes after THA in patients with prior arthroscopy, with increased reoperation and dislocation [7], increased revision [11, 17], and increased infection and aseptic loosening [11]. These findings appeared strongest when THA was performed within 1 year of arthroscopy [7] and when the prior arthroscopy had been undertaken for osteoarthritis [11]. This discrepancy in postoperative complications and revision-related events is unlikely to be explained by chance alone. A short interval between arthroscopy and THA may reflect failed joint-preservation treatment in a hip that was already progressing toward arthroplasty, rather than an isolated effect of the arthroscopic procedure itself. In this context, prior arthroscopy should be interpreted not only as a surgical history variable, but also as a potential marker of pre-existing joint degeneration or suboptimal indication for preservation surgery, both of which may contribute to a higher risk of postoperative complications and revision-related events. The subgroup at potentially higher risk can therefore be described as patients who underwent arthroscopy in the presence of established osteoarthritis or rapidly progressive degenerative disease. In these cases, prior arthroscopy may represent confounding by indication: patients selected for arthroscopy before THA may differ from standard primary THA patients in age, activity level, baseline morphology, cartilage status, disease trajectory, and expectations. Accordingly, the observed associations should not be interpreted as definitive evidence that arthroscopy itself causes inferior THA outcomes, but rather as evidence that failed joint-preservation surgery in degenerative hips may identify a more complex clinical subgroup.

Functional outcomes were generally less concerning than complication outcomes. In most studies, THA was associated with substantial postoperative improvement irrespective of prior arthroscopy, and no significant between-group differences were found in mHHS, WOMAC, or UCLA activity score [9], postoperative HHS [6], EQ-5D, EQ VAS, hip pain, or satisfaction at 1 year [10], and no significant differences in mid-term patient-reported outcomes in another study [17]. However, slightly inferior postoperative scores were reported in some studies, including lower HOOS and WOMAC domains and lower SF-12 physical scores [16], lower HHS, FJS-12, and satisfaction [13], and lower Oxford Hip Score [20]. These differences were not consistently reproduced across studies and were generally modest in magnitude, suggesting that prior arthroscopy does not negate the functional benefit of THA, although it may be associated with slightly less favorable patient-reported recovery in selected patients. Importantly, statistically significant differences in PROMs should not automatically be interpreted as clinically meaningful differences. Most included studies did not report MCID, PASS, or other responder-based thresholds, which limits interpretation of whether the observed between-group differences were meaningful to patients. Therefore, the available PROM evidence supports the conclusion that THA generally provides substantial functional improvement after prior arthroscopy, while the clinical importance of between-group score differences remains uncertain.

These findings must be interpreted in light of the limitations of both the included literature and this review. All included studies were retrospective and exposed to selection bias, residual confounding, heterogeneous matching strategies, and non-standardized reporting of arthroscopy indications, techniques, and outcome measures. In addition, not all studies clearly separated arthroscopy performed for femoroacetabular impingement from arthroscopy performed in the setting of osteoarthritis, and patient-reported outcome reporting was not fully uniform. These limitations reduce precision, but they do not invalidate the main review finding because the same broad pattern recurs across different study designs and secondary syntheses. The review protocol was not prospectively registered in PROSPERO, which should be acknowledged as a limitation. Large administrative database studies also carry specific limitations, including coding error, limited clinical granularity, inability to verify arthroscopy indications or operative details, and possible overlap of patient populations across databases.

## Conclusion

Prior ipsilateral hip arthroscopy does not appear to preclude satisfactory results after subsequent total hip arthroplasty. In most comparative studies, perioperative differences were limited and postoperative functional improvement remained substantial. Evidence regarding postoperative complications and revision-related events was heterogeneous, although some studies reported increased risk in selected situations, particularly when THA followed arthroscopy after a short interval or when arthroscopy had been performed in an already degenerative hip. Overall, these findings support a cautious interpretation, prior arthroscopy should not be viewed as a contraindication to successful THA, but attention should be paid to indication and timing, especially in osteoarthritic hips.

## Data Availability

All data generated and/or analysed during the current study are included in this article and its tables. Additional extracted data are available from the corresponding author on reasonable request.

## Abbreviations

A+: Prior arthroscopy group
A−: No prior arthroscopy group
BMI: Body mass index
EQ-5D: EuroQol 5-Dimension questionnaire
EQ VAS: EuroQol visual analogue scale
FAI: Femoroacetabular impingement
FJS-12: Forgotten Joint Score-12
HHS: Harris Hip Score
HOOS: Hip disability and Osteoarthritis Outcome Score
HRA: Hip resurfacing arthroplasty
LEAS: Lower Extremity Activity Scale
mHHS: Modified Harris Hip Score
MINORS: Methodological Index for Non-Randomized Studies
NA: Not available / not applicable / not specified
OHS: Oxford Hip Score
PICOS: Population, Intervention, Comparator, Outcomes, and Study design
PJI: Periprosthetic joint infection
PRISMA: Preferred Reporting Items for Systematic Reviews and Meta-Analyses
PRO: Patient-reported outcome
PROM: Patient-reported outcome measure
QoL: Quality of life
RR: Relative risk
SF-12: 12-item Short Form Health Survey
SSI: Surgical site infection
THA: Total hip arthroplasty
THR: Total hip replacement
UCLA: University of California, Los Angeles activity score
VAS: Visual analogue scale
VR-12: Veterans RAND 12-item Health Survey
WOMAC: Western Ontario and McMaster Universities Osteoarthritis Index
